# Peak Outpatient and Emergency Department Visit Forecasting for Patients With Chronic Respiratory Diseases Using Machine Learning Methods: Retrospective Cohort Study

**DOI:** 10.2196/13075

**Published:** 2020-03-30

**Authors:** Junfeng Peng, Chuan Chen, Mi Zhou, Xiaohua Xie, Yuqi Zhou, Ching-Hsing Luo

**Affiliations:** 1 School of Data and Computer Science Sun Yat-sen University Guangzhou China; 2 Surgical Intensive Care Unit The Third Affiliated Hospital of Sun Yat-sen University Guangzhou China; 3 Department of Respiratory and Critical Care Medicine The Third Affiliated Hospital of Sun Yat-sen University Guangzhou China

**Keywords:** chronic respiratory diseases, ensemble machine learning, health forecasting, outpatient and emergency departments management

## Abstract

**Background:**

The overcrowding of hospital outpatient and emergency departments (OEDs) due to chronic respiratory diseases in certain weather or under certain environmental pollution conditions results in the degradation in quality of medical care, and even limits its availability.

**Objective:**

To help OED managers to schedule medical resource allocation during times of excessive health care demands after short-term fluctuations in air pollution and weather, we employed machine learning (ML) methods to predict the peak OED arrivals of patients with chronic respiratory diseases.

**Methods:**

In this paper, we first identified 13,218 visits from patients with chronic respiratory diseases to OEDs in hospitals from January 1, 2016, to December 31, 2017. Then, we divided the data into three datasets: weather-based visits, air quality-based visits, and weather air quality-based visits. Finally, we developed ML methods to predict the peak event (peak demand days) of patients with chronic respiratory diseases (eg, asthma, respiratory infection, and chronic obstructive pulmonary disease) visiting OEDs on the three weather data and environmental pollution datasets in Guangzhou, China.

**Results:**

The adaptive boosting-based neural networks, tree bag, and random forest achieved the biggest receiver operating characteristic area under the curve, 0.698, 0.714, and 0.809, on the air quality dataset, the weather dataset, and weather air quality dataset, respectively. Overall, random forests reached the best classification prediction performance.

**Conclusions:**

The proposed ML methods may act as a useful tool to adapt medical services in advance by predicting the peak of OED arrivals. Further, the developed ML methods are generic enough to cope with similar medical scenarios, provided that the data is available.

## Introduction

Worldwide, one of the fundamental issues in hospital management is the sudden inflow of outpatient and emergency department (OED) patients [1]. Influenza season (epidemic period) is one of the causes for OED overcrowding and generates a large flow of patients [2]. In particular, weather and air quality are important factors that affect the health status of individuals and populations with chronic respiratory diseases [3]. Chronic respiratory diseases such as asthma and chronic obstructive pulmonary disease (COPD) often require regular OED medication as the condition changes, which can cause further OED overcrowding [4]. Nevertheless, the crowding could be alleviated and mitigated considerably by forecasting levels of demand for OED care and giving health care staff an opportunity to prepare for this demand [5]. Efficient patient flow has been proven to potentially increase the capacity of the existing system, minimize patient care delays, and improve overall quality of health care [6-10].

There have been many attempts to predict daily patient volumes visiting emergency departments (EDs) using machine learning (ML) and deep learning models based on weather and air quality [11,12].

Bibi et al [13] created a computer-based model called an artificial neural network (ANN) using a backpropagation to predict volumes of ED visits of patients with asthma, COPD, or acute or chronic bronchitis 7 days in advance. The study included a dataset (1020 days of ED activity) extracted from an ED admittance database at the Barzilai Medical Center (Ashkelon, Israel). The mode integrated 5 indicators (ie, temperature, relative humidity, barometric pressure, sulfur dioxide, and nitrogen oxide) and achieved the prediction accuracy with an average error of 12%. However, indicators and data collections are relatively inadequate.

Moustris et al [14] developed three different ANN models to forecast the childhood asthma admissions 7 days in advance for the subgroups of 0 to 4 years of age and 5 to 14 years of age, as well as for the whole study population. The study used 6 indicators, that is ozone, carbon monoxide, PM10 (particulate matter of 10 μm in diameter or smaller), PM25 (particulate matter less than 2.5 μm in diameter), and sulfur dioxide, from Athens, Greece to train the ANN model. The evaluation of the three ANN models’ forecasting abilities on the root mean square error (mean bias error) were 6.8 (1.4), 3.2 (1.3), and 5.2 (0.3) for 0 to 4 years of age, 5 to 14 years of age, and the whole study population, respectively. However, the study only took into account air quality indicators and ignored weather factors.

Soyiri [15] explored the base and reduced predictive quantile regression models (QRMs) to detect peak numbers of daily asthma admissions in London with sensitivity levels of 76% and 62%; as well as specificities of 66% and 76%, respectively. The research used 10 indicators (ie, air temperature, vapor pressure, humidity, ozone, carbon monoxide, nitrogen dioxide, nitrogen oxide, PM10, and formaldehyde) to build the QRMs. The findings also reaffirmed the known associations between asthma and temperature, and ozone and carbon monoxide levels. Nevertheless, the accuracy of the model is not very high.

Khatri et al [16] employed an ANN–based classifier using multilayered perceptions with a backpropagation algorithm that predicts peak events, that is days of peak demand, for patients with respiratory diseases. The study used 8 predictors (ie, outdoor temperature, relative humidity, wind speed, carbon monoxide, ozone, sulphur dioxide, nitrogen dioxide, and PM25) to construct the model. The proposed ANN model achieved a good forecasting performance with the overall accuracy of the system at 81.0%. Even so, the study population only included visits for respiratory diseases data in EDs. Further, the research did not consider dividing data into weather and air pollution separately.

Yucesan et al [17] developed a multi-method patient arrival forecasting outline for EDs using a private hospital ED case in Turkey. The methods followed within this study include the single methods linear regression (LR), autoregressive integrated moving average (ARIMA), ANN, exponential smoothing, and the hybrid methods ARIMA-ANN and ARIMA-LR. The ARIMA-ANN hybrid model is shown to outperform in terms of forecasting accuracy. This study explored a novel attempt of applying these methods to model ED patient arrivals and making an overall assessment among them.

Muhammet et al [18] analyzed variations in annual, monthly, and daily ED arrivals based on regression and neural network models with the aid of collected data from a public hospital ED in Istanbul. Both of the methods have been proven to be useful and readily available tools for forecasting ED patient arrivals. The results show that ANN–based models have higher model accuracy values and lower values of absolute error in terms of forecasting ED patient arrivals over the long- and medium-term. The value of the standard error of regression for the ANN modeling, which is 30.022306, refers to the difference between the real ED patient arrivals and the forecasted ED patient arrivals per day covering the total of the three patient groups.

Although ED forecasting has attracted many researchers, we found few studies designed to predict OED visits of patients with chronic respiratory diseases using multiple ML methods. In a real medical scene, patients with chronic respiratory diseases often go to outpatient clinics. Therefore, it would be of great significance to forecast the peak OED visits for chronic respiratory diseases.

In this paper, we employed bagging [19], adaptive boosting [20] and random forest [21] algorithms to predict the peak arrival of chronic respiratory disease OED visits based on the weather and air quality data. Meanwhile, we compared the ensemble models with the general linear model (GLM) [22] and the polynomial nuclear support vector machine (SVM) [23]. The results show that ensemble models outperform the GLMs and SVM. Further, we found that the predictive performance of ML algorithms gradually improves with the increase of input features. By the ML approaches, the OED managers can plan resources to meet the excessive demand of patients with respiratory diseases after short-term fluctuations in air pollution or weather.

## Methods

### Data Acquisition

**Figure 1:**
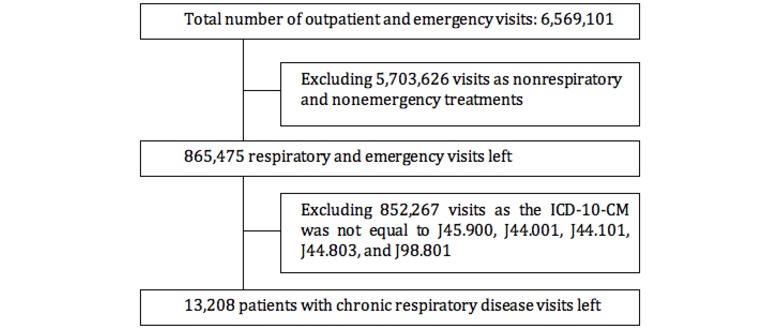
Flowchart of participants. ICD-10-CM: International Classification of Diseases, 10th revision, Clinical Modification.

Figure 1 shows the flowchart of participants in our research. We identified 13,208 OED visits to the Second Affiliated Hospital of Guangzhou Medical University that had a major diagnosis of a chronic respiratory disease defined by the International Classification of Diseases, Tenth Revision, Clinical Modification code (J45.900, J44.001, J44.101, J44.803, and J98.801). The duration of the collected data lasted from January 1, 2016, to December 31, 2017, which is 731 days of continuous data. For statistical purposes, the days where the daily volume was less than 24 were labeled as nonpeak events, and the rest were labeled as peak events.

Table 1 describes the Pearson correlation coefficient between OED visit numbers and input indicators. We found that OED visit numbers showed positive correlations with wind speed, atmospheric pressure, carbon monoxide, sulphur dioxide, nitrogen dioxide, and PM25. However, OED visit numbers showed negative correlations with outdoor temperature, relative humidity, and ozone. The weather and air quality data distribution of patients with acute exacerbations of COPD from peak and nonpeak groups was shown in Table 2.

**Table 1:** The Pearson correlation coefficients between outpatient and emergency department visit numbers and input indicators.

Variable	WS^a^, r	TP^b^, r	AP^c^, r	RH^d^, r	PM25^e^, r	SO_2_^f^, r	CO^g^, r	NO_2_^h^, r	O_3__8h^i^, r	Number of visits, r
WS	1	–0.32	0.27	–0.4	–0.34	–0.33	–0.26	–0.42	–0.24	0.15
TP	–0.32	1	–0.88	0.35	–0.23	0.03	–0.24	–0.25	0.39	–0.38
AP	0.27	–0.88	1	–0.5	0.31	0.09	0.21	0.29	–0.18	0.39
RH	–0.4	0.35	–0.5	1	–0.18	–0.27	0.2	0.03	–0.28	–0.2
PM25	–0.34	–0.23	0.31	–0.18	1	0.73	0.65	0.81	0.29	0.29
SO_2_	–0.33	0.03	0.09	–0.27	0.73	1	0.35	0.66	0.43	0.22
CO	–0.26	–0.24	0.21	0.21	0.65	0.35	1	0.68	–0.07	0.35
NO_2_	–0.42	–0.25	0.29	0.03	0.81	0.66	0.68	1	0.13	0.35
O_3__8h	–0.24	0.39	–0.18	–0.28	0.29	0.43	–0.07	0.13	1	–0.14
Number of visits	0.15	–0.38	0.39	–0.2	0.29	0.22	0.35	0.35	–0.14	1

^a^WS: wind speed.

^b^TP: outside temperature.

^c^AP: atmospheric pressure.

^d^RH: relative humidity.

^e^PM25: particulate matter less than 2.5 μm in diameter.

^f^SO_2_: sulphur dioxide.

^g^CO: carbon monoxide.

^h^NO_2_: nitrogen dioxide.

^i^O_3__8h: 8-hour average ozone slip in a day.

**Table 2:** Weather and air quality data distribution of peak and nonpeak groups visiting outpatient and emergency departments.

Variables	Peak group, mean (SD)	Nonpeak group, mean (SD)
Wind speed (m/sec)	2.49 (1.10)	2.15 (0.91)
Outside temperature (°C)	17.81 (5.59)	23.11 (5.81)
Atmosphere pressure (mb)	1009.99 (5.26)	1003.73 (6.57)
Relative humidity (%)	77 (12.51)	82.15 (9.65)
Particulate matter less than 2.5 μm in diameter	43.74 (23.69)	32.83 (16.49)
Sulphur dioxide	13.16 (4.65)	11.45 (3.73)
Carbon monoxide	1.06 (0.25)	0.92 (0.17)
Nitrogen dioxide	60.05 (26.09)	46.43 (17.67)
8-hour average ozone slip in a day	74.28 (54.90)	90.24 (52.46)

### Data Analysis

Since the effect of weather and air quality on respiratory conditions in humans was not instantaneous, representative lags were applied to variables based on the work done previously in this area [3,24-26]. To simplify the delayed impact of respiratory conditions, we considered a 3-day lag for all variables.

We removed records with less than 10 people on weekends to eliminate weekend effects, bringing the total number of samples collected to 559. To create a meaningful feature vector for training and cross-validation, the date field was removed to obtain a (*X*, *y*), where *X* was a matrix with the dimensions (*m* × *n* = 559 × 9) representing values of variables, and *y* was a vector of length (m=559) representing the output class of the examples (ie, events). Analysis of the data suggested that the output class was highly imbalanced with 413 examples of nonpeak and 146 examples of peak events.

### Machine Learning Approaches

In this section the ML algorithms are presented and discussed; details of the updating and classification processes are described in the following algorithms.

#### Generalized Linear Models

1. Construct the common linear model from the original training set: *f* 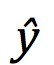 = *w^T^ x* + *b*, where *w* is the weight vector and *b* is the bias, both of which are only determined by the training samples
2. Identify the contact function *f* ^-1^
3. Build the GLMs: 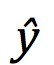 = *f* ^-1^ (*w^T^ x* + *b*)
4. Calculate the classification on the test set

#### Support Vector Machine

1. Convert the sample space into linearly separable space with polynomial core functions *K* (*x*_i_, *y*_i_)
2. Calculate the support vectors with the following formula: 
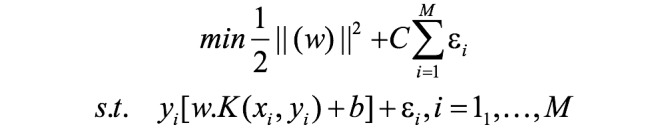
3. Then identify the hyperplane. The regular parameter *C* is a penalty factor, which can balance the model complexity and empirical risk. In addition, ε*_i_* indicates the positive parameters called slack variables, which represent the distance between the misclassified sample and the optimal hyperplane.
4. Forecast the classification of the test dataset using hyperplane and support vectors

#### Bagging

1. Generate a new training set by sampling from the original training set
2. Repeat step 1 N times to get the N new training sets, and train N trees in N different training sets
3. Calculate the classification results by averaging the predicted value of each tree or use the majority
4. Out-of-bag error estimation: The data not sampled in step 1 is used as the test set of the corresponding generated tree to evaluate the predicted results

#### Random Forest

1. Create a new training set from a sample of a training set
2. Repeat step 1 N times to get N new training sets, and train N trees on the training sets
3. Identify the optimal candidate node as the prediction space from the randomly selected m feature set when building the tree model

#### Boosting

1. Initialize the weight vector of the training data
2. Construct m weak classifiers
3. Calculate the classification error rate of the m weak classifiers
4. If one sample is misclassified, its weight will be increased, and the next weak classifier pays more attention to this sample; otherwise, its weight will be decreased.
5. After all the weak classifiers finish the training, the stronger classifier is constructed.

## Results

### Metrics

Precision, recall, and F measure are the metrics that are used to evaluate our proposed ML methods. Based on the classification of true positives (TP), false positives (FP), true negatives (TN), and false negatives (FN), we have the following formulas.


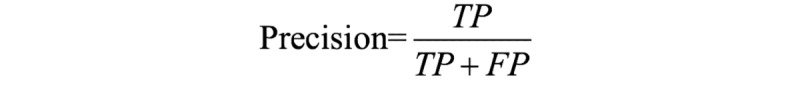



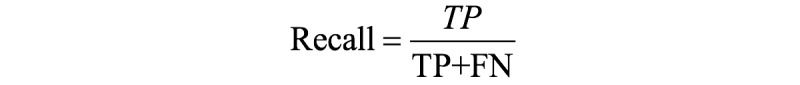


We then define the F measure, a metric that balances precision and recall.


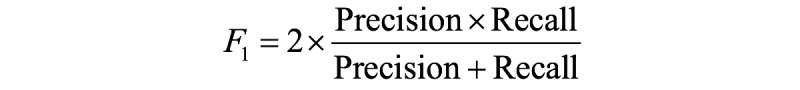



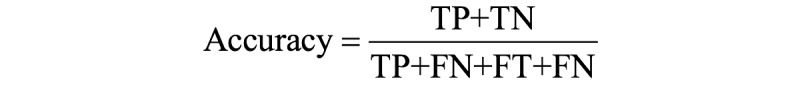


### Evaluation

We calculate the overall accuracy, precision, recall, and F measure for nonpeak events and peak events, respectively. Evaluation of the ML approaches on the weather and air quality data are shown in Table 3. It showed that the developed random forest gave the best predictive performance. This was mainly due to the data collection fitting better with the random forest.

**Table 3:** Evaluation of machine learning approaches on weather and air quality.

Machine learning approaches		F1 measure	Accuracy, % (n/N)
**Generalized linear model**			85.6 (479/559)
	Peak	0.667	
	Nonpeak	0.908	
**Support vector machine**			80.2 (448/559)
	Peak	0.289	
	Nonpeak	0.882	
**Adaptive boosting neural networks**			84.7 (473/559)
	Peak	0.667	
	Nonpeak	0.900	
**Tree bag**			83.8 (468/559)
	Peak	0.640	
	Nonpeak	0.895	
**Random forest**			88.3 (494/559)
	Peak	0.745	
	Nonpeak	0.924	

In addition, we used the receiver operating characteristic (ROC) curve to evaluate the multiple ML approaches on the same dataset (Table 4). We found that adaptive boosting neural networks achieved the biggest ROC area under the curve on the air quality data, tree bag on the climate data, and random forest on weather and air quality data. In general, we discovered that the predictive performance of the ML approaches improves as data variables increase.

**Table 4:** Evaluation of machine learning approaches using receiver operating characteristic.

Machine learning approaches	Weather, AUC^a^	Air quality, AUC	Weather and air quality, AUC
Generalized linear model	0.538	0.682	0.758
Support vector machine	0.500	0.494	0.621
Adaptive boosting neural network	0.611	0.698	0.734
Tree bag	0.714	0.680	0.780
Random forest	0.669	0.692	0.809

^a^AUC: area under the curve.

## Discussion

### Clinical Significance

**Figure 2:**
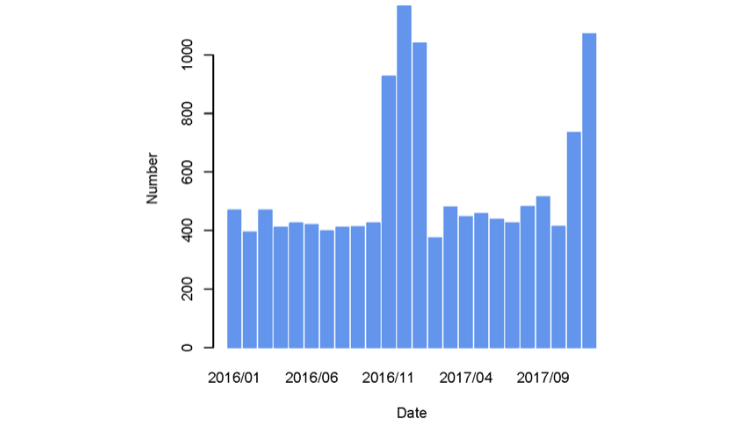
Histogram of patients visiting outpatient and emergency rooms.

Recent studies have shown that weather and air pollution have been a major problem leading to an increase in daily deaths and hospital admissions for chronic respiratory illnesses [3-5,27,28]. We focused the distribution of daily patient visits for 2 years (ie, 2016 and 2017) (Figure 2). It is worth noting that peak days are more dominant from October to March, which indicates that the haze is a strong predictor, as these months are mostly colder in Guangzhou. Thus, it is important to recognize the peak OED visits for respiratory conditions.

Previous studies mainly focused on the peak event forecasting ED visits for patients with one or more diseases. We expanded the study population to include outpatient visits for patients with chronic respiratory diseases. In fact, many patients with chronic respiratory diseases also seek treatment from outpatient departments. Thus, predicting the OED peak visits for chronic respiratory disease plays an important role in clinical management.

We developed a variety of learning methods to forecast the OED peak visits, from simple models to complex ensemble learning ones. In particular, the ensemble learning models achieved good prediction results. In terms of indicators, most of the previous studies used air pollution indicators to predict the peak events of ED visits; however, we used weather and air quality indicators to build a more complete set of features.

### Limitations

There are a few limitations to this study. In this study, we used nine variables, namely, wind speed, atmospheric pressure, outdoor temperature, relative humidity, carbon monoxide, ozone, sulphur dioxide, nitrogen dioxide, and PM25, as these variables have been associated with exacerbation of respiratory diseases. However, there are some other variables that also contribute to the exacerbation of these diseases, such as formaldehyde and nitrogen oxide [29]. The Environmental Protection Agency of Guangzhou does not disclose the daily data for variables such as formaldehyde. Other pollutants are either not measured or had too many missing values. Therefore, we were not able to include these variables in our study.

In terms of weather, Guangzhou as a coastal city in southern China has a higher air humidity than other northern cities. In terms of air pollution, some studies have shown that patients with lower levels of economics and education are more susceptible to air pollution [30]. Guangzhou has a significantly higher economic and educational level than the national average. However, the pollution of haze and the harmful emissions of Guangzhou are also serious [31]. In particular, the lighter particulate matter is higher than other northern cities due to automobile exhaust and industrial emissions. Therefore, the prediction result of this study may not be directly applicable to other regions due to the regional differences in climate and air pollution.

### Conclusion

In this paper, we investigated ML methods to forecast the peak events of patients with chronic respiratory diseases visiting OEDs combined with nine weather and air quality predictors. Overall, random forest outperforms the other methods in the accuracy, F measure, and ROC on the validation dataset. Compared with similar studies before, we used more indicators and ML methods to study the subject and achieved good results. The ML methods may act as a useful tool to adapt medical services in advance by predicting the peak number of OED arrivals.

## Abbreviations

ANN: artificial neural network
ARIMA: autoregressive integrated moving average
COPD: chronic obstructive pulmonary disease
ED: emergency department
FN: false negatives
FP: false positives
GLM: general linear model
LR: linear regression
ML: machine learning
OED: outpatient and emergency department
PM10: particulate matter of 10 μm in diameter or smaller
PM25: particulate matter less than 2.5 μm in diameter
QRM: quantile regression model
ROC: receiver operating characteristic curve
SVM: support vector machine
TN: true negatives
TP: true positives
